# Afro-TB dataset as a large scale genomic data of ***Mycobacterium tuberuclosis*** in Africa

**DOI:** 10.1038/s41597-023-02112-3

**Published:** 2023-04-14

**Authors:** Meriem Laamarti, Yasmine El Fathi Lalaoui, Rachid Elfermi, Rachid Daoud, Achraf El Allali

**Affiliations:** African Genome Center, Mohammed VI Polytechnic University, Ben Guerir, 43150 Morocco

**Keywords:** Pathogens, Computational biology and bioinformatics

## Abstract

*Mycobacterium tuberculosis* (MTB) is a pathogenic bacterium accountable for 10.6 million new infections with tuberculosis (TB) in 2021. The fact that the genetic sequences of *M. tuberculosis* vary widely provides a basis for understanding how this bacterium causes disease, how the immune system responds to it, how it has evolved over time, and how it is distributed geographically. However, despite extensive research efforts, the evolution and transmission of MTB in Africa remain poorly understood. In this study, we used 17,641 strains from 26 countries to create the first curated African *Mycobacterium tuberculosis* (MTB) classification and resistance dataset, containing 13,753 strains. We identified 157 mutations in 12 genes associated with resistance and additional new mutations potentially associated with resistance. The resistance profile was used to classify strains. We also performed a phylogenetic classification of each isolate and prepared the data in a format that can be used for phylogenetic and comparative analysis of tuberculosis worldwide. These genomic data will extend current information for comparative genomic studies to understand the mechanisms and evolution of MTB drug resistance.

## Background & Summary

Tuberculosis (TB) remains one of the deadliest contagious diseases caused by the *Mycobacterium tuberculosis* complex (MTBC). According to the World Health Organization’s TB 2021 report, COVID-19 has reversed years of global success in the fight against tuberculosis. At 9.9 million cases in 2020, the number of tuberculosis deaths increased for the first time in more than a decade^1^. The increase in TB deaths occurred primarily in the 30 countries with the highest burden of TB, which mainly include countries from Africa^1^. In many other African countries, WHO estimates that many people now have tuberculosis but have not been diagnosed or officially reported to national authorities^1,2^. In Morocco, for example, the 2020 report captured only TB-HIV cases and reported the highest number of deaths among TB-HIV negative cases in the past 20 years. Similarly, TB mortality in South Africa increased in 2020 and is expected to continue to increase over the next five years^1^.

Africa is the only continent that harbors all MTBC lineages, and it has been hypothesized to be the origin of this pathogen^3^. Under this hypothesis, characterizing the genetic diversity of MTBC strains detected in Africa is important for understanding the spread and evolution of antibacterial resistance of TB. Multidrug-resistant *Mycobacterium tuberculosis* (MDR-TB) is a major threat to global TB control strategies. In 2017, 26,845 MDR, Rifampicin resistant TB (RR-TB) and 867 extensively drug resistant TB (XDR-TB) cases were reported in Africa^1^. The increasing detection of drug-resistant TB has raised concern and motivated stricter surveillance and control measures to prevent further escalation of drug resistance. Intensive research has been conducted to decipher the resistance mechanisms and drug resistance profiles of TB in Africa,^4,5^.

**Fig. 1:**
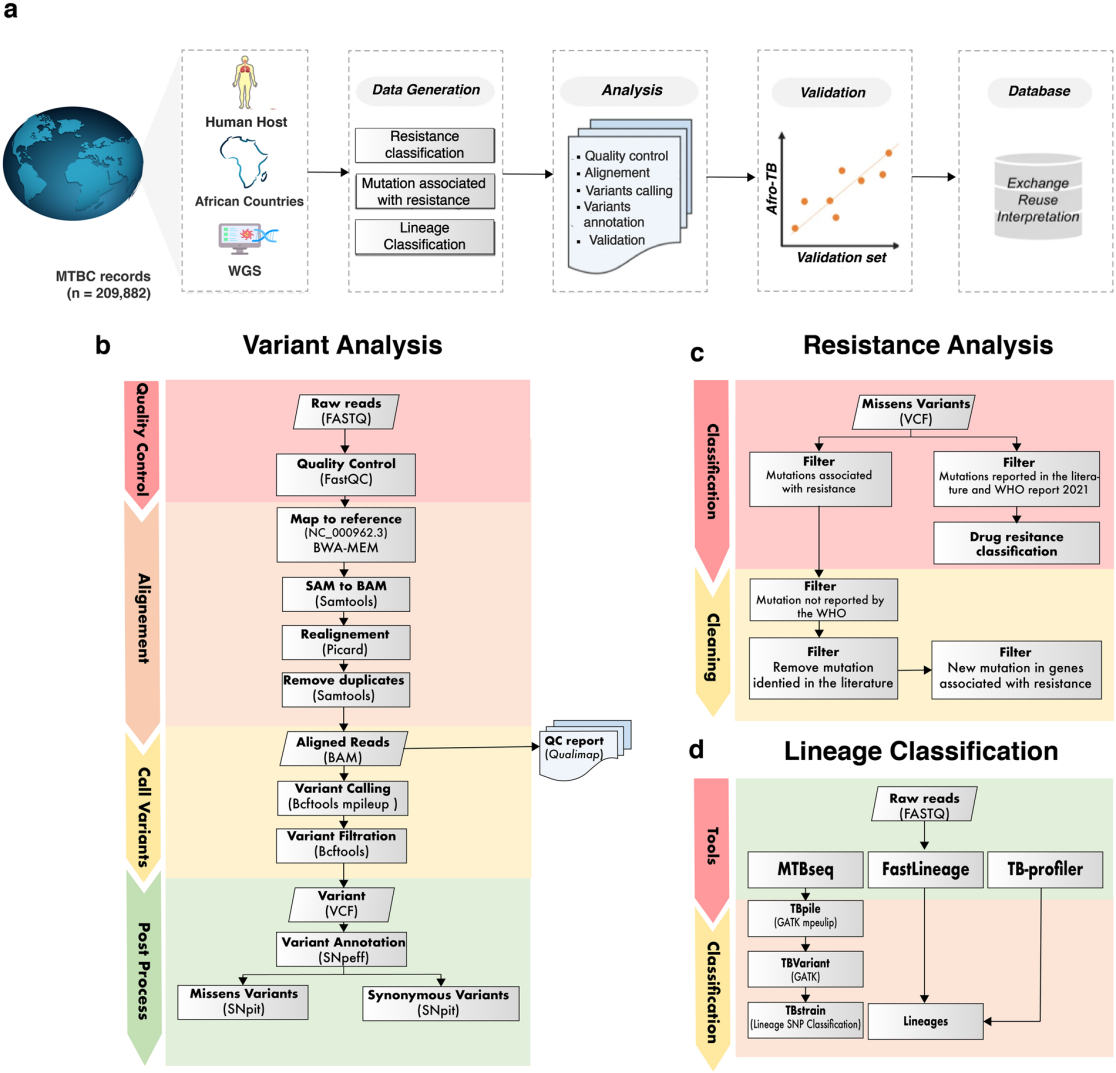
Flowchart of the Afro-TB Workflow: (**a**) data collection and processing pipeline, (**b**) variant analysis pipeline, (**c**) resistance pipeline, and (**d**) classification pipelines.

The fields of bioinformatics and genomics have already had a major impact on public health by helping researchers track the spread of TB and predict whether individual patients will develop resistance to TB drugs^6,7^. To compensate for the delay in the TB strategy forced by COVID-19, WHO is focusing on global action against TB in African countries where progress is most needed. afro. Currently, several databases are available that have been created by studying the correlation between phenotype and genotype data worldwide^8,9^. These databases serve as a reference point for identifying drug resistance mutations and help researchers collect the data needed for TB research. Other comprehensive databases such as TB Database and TubercuList provide information on TB genes and proteins, but are no longer updated^10,11^. The TBrowse database, on the other hand, allows users to visualize and analyze the genome sequence of *M. tuberculosis*, but these databases only provide information on structural variations and resistance^12^. The largest TB database currently available is SRA TB-profiler, which contains 16,000 strains and provides information on resistance and lineages, but it covers only 8,000 strains from Africa and its results cannot be downloaded for further analysis^13^. In line with the goal of WHO and in an effort to provide the research community with a large African tuberculosis dataset with high-quality data we created The Afro-TB. In this Data Descriptor, we report a rigorous dataset (AFRO-TB) extracted from 13,753 collected genomes of *Mycobacterium tuberculosis* from human hosts in 26 African countries and analyzed with more than 20,000 CPU hours on high-memory machines and more than 50 TB of storage. We performed quality control (QC) to ensure the quality of paired-end whole genome sequencing data. These data were analyzed to identify resistance mutations and lineages circulating in Africa. In addition, we compared the extracted genome with previously published resistance-associated mutations in *M. tuberculosis* and with mutations published by WHO in 2021 using more than 120 resistance-associated genes (https://www.who.int/publications-detail-redirect/9789240028173). Variant calling and lineage classification proved to be excellent tools for phylogenetic tree analysis. Figure 1 shows the study design and how the data were collected.

A list of the number of samples collected from each country can be found in Table 1. To our knowledge, AFRO-TB is currently the largest public dataset for drug resistance and lineage classification, providing researchers with flexible searching and immediately usable results to help them study tuberculosis more effectively.

**Table 1:** Number of samples in each country.

Country	Number of samples
Algeria	88
Botswana	94
Cameroon	199
Ivory cost	62
Congo	562
Djibouti	180
Eswatini	51
Ethiopia	288
Gambia	465
Ghana	272
Guinea	185
Kenya	40
Liberia	42
Madagascar	163
Malawi	1789
Mali	279
Mozambique	77
Nigeria	141
Rwanda	417
Sierra Leone	134
South Africa	6902
Sudan	158
Tanzania	550
Tunisia	100
Uganda	490
Zimbabwe	25

## Methods

### Data collection and selection

We conducted a search on the NCBI database for metadata related to *M.tuberculosis* up until September 20, 2022, without any limitations on the geographic location. This search was performed using the NCBImeta tools^14^ with customized configuration. Values for the query parameters were [Assembly: (tuberculosis OR *Mycobacterium tuberculosis*), BioProject: (tuberculosis OR *Mycobacterium tuberculosis*) AND (bioproject assembly[Filter] OR bioproject sra[Filter]), BioSample: (tuberculosis OR *Mycobacterium tuberculosis*) AND (biosample assembly[Filter] OR biosample sra[Filter]), SRA: ((tuberculosis OR *Mycobacterium tuberculosis*) AND (genome OR genomes OR genomic OR genomics) NOT transcriptomic[Source]) Fig. 1. Data compression and decompression was done using MZPAQ^15^.

We compiled the whole-genome sequence (WGS) metadata collection of *M.tuberculosis* isolates exclusively from Africa. Of the more than 120,000 unique results, only data that met the following criteria were used: i) strains isolated from human hosts; ii) strains from African countries; iii) whole-genome sequencing data; iv) strains with less than 10% contamination.

The SRA accession numbers of more than 17,000 paired-end files were downloaded from the NCBI Sequence Read Archive (SRA) (http://ncbi.nlm.nih.gov/sra) using fastq-dump. The fastq data were quality checked using FastQC and 15,384 isolates were retained^16^. Kraken2^17^ was used to identify the percentage of reads not belonging to the *Mycobacterium tuberculosis* complex and to remove highly contaminated genomes, resulting in 13,753 isolates. A complete list of accession numbers of the selected genomes with their distribution by country and collection date can be found in the dataset **(AFRO-TB Dataset Accession-numbers)**^18^.

### Variants calling

Paired-end short reads were trimmed for quality using trimmomatic v 0.39^19^ (sliding-window trimming with a window size of 4 and a read quality threshold of 30) and all ambiguous sequences were eliminated to exclude mixed samples. The processed short reads were mapped to the *M. tuberculosis H37Rv* reference genome (NC_000962.3) using *bwa mem* for paired-end^20^. The bam file was sorted using samtools. We removed sequencing reads with an average sequencing coverage depth >20x using bedtools. We then looked for PCR duplicates that should be removed as this helps to reduce the number of artifactual variants in low-frequency regions. Duplicate reads were masked using MarkDuplicates from Picard (http://broadinstitute.github.io/picard/http://broadinstitute.github.io/picard/) and variants were called using Bcftools v4.1.6.0^21^ (base quality score ≥20, haploid model). Bcftools was run with the parameters “-T HaplotypeCaller -R ref.fasta -I sample.bam -o sample.vcf-min-base-quality-score 20 -ploidy 1”. Variants annotation was performed with SnpEff after building the SnpEff database using the *M. tuberculosis H37Rv* reference genome (NC_000962.3)^22^ (Fig. 1a).

### Lineage analysis

Lineage classification is based on the identification of specific single nucleotide polymorphisms (SNPs) associated with different branches of the evolutionary tree of the bacterium^23–25^. The result of this analysis is a file showing which lineage each sample belongs to, along with an indication of how confident the classification is, based on the quality of the data at the positions used for the analysis. This step was performed using a tool called Fastlineage v1.0 (https://github.com/farhat-lab/fast-lineage-caller) and only lineages reported by more than one database were considered. Lineage classification is based on a set of phylogenetic SNPs^23–25^. The output is a classification file with the reported lineage for each record. The file also gives an indication of the quality of the data for the positions used to infer the phylogenetic classification.

### Resistance analysis

To identify the mutations associated with resistance, we compared the variants obtained in the VCF files with the published mutations and their associated antibiotic. All mutations associated with resistance according to WHO and the literature were used as reference for resistance identification **(AFRO-TB Dataset WHO-resistance-associated-mutations)**^18^. These mutations were identified in our data and used for the resistance profile classification. Based on the mutation results, we classified the analyzed *Mycobacterium tuebrculosis* strains into 5 categories: Susceptible [no mutation associated with resistance], Monoresistant (Mono) [Isoniazid or Rifampicin], MDR [Rifampicin and Isoniazid], PreXDR [Rifampicin and Isoniazid plus Fluoroquinolones], XDR [Rifampicin and Isoniazid plus Fluoroquinolones and at least one of the second-line drugs (Kanamycin, Capreomycin, or Amikacin)] **(AFRO-TB Dataset Lineage-drug-resitance-classifiation)**^18^. To identify new mutations, we discarded all mutations present in the WHO report and in the literature **(AFRO-TB Dataset Lineage-drug-resitance-classifiation)**^18^. The new mutations were considered potentially associated with resistance but require further analysis **(AFRO-TB Dataset Undescribed-mutations)**^18^).

## Data Records

**Fig. 2:**
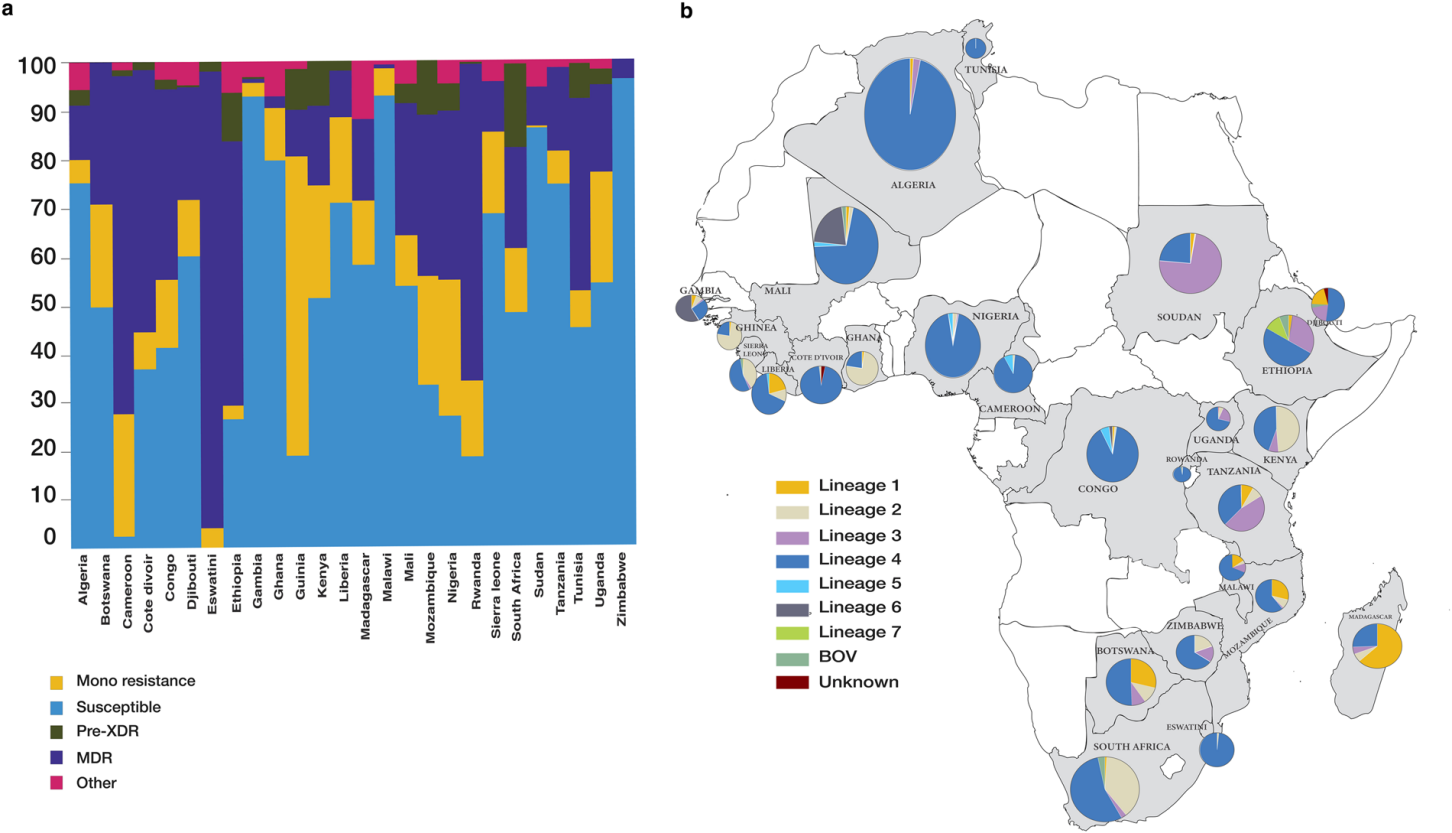
*Mycobacterium tuberculosis* distribution in 23 African countries. (**a**) The graph depicts the distribution of MTB resistance profiles in each country. (**b**) The pie charts depict the MTB lineage distribution in each country.

The datasets are suitable for different drug resistance and phylogenetic analysis pipelines as they provide data from 26 countries in Africa. The distribution of lineages and drug resistance in each country are included in the dataset to facilitate comparison with other cases of *M. tuberculosis* worldwide (Fig. 2).

The Afro-TB dataset includes three sets of files: (1) VCF files annotated with the reference genome “*Mycobacterium tuberculosis H37Rv*”. Each VCF file represents a sample containing all mutations, their genomic and proteomic positions, and the genes that harbor them. (2) A filtered file in tabular format containing the positions of the mutations in the reference genome, genes and proteins. (3) A metadata table containing information about the strains (country of origin, lineage classification, and drug classification). This table also contains all mutations associated with resistance and their antibiotic associations for each isolate, as well as the corresponding VCF files. We deposited the dataset as a Figshare repository^18^ and made it dynamically available https://bioinformatics.um6p.ma/AfroTB/. Researchers can search the dataset by country, lineage, resistance, or drug. They can also submit new samples, which will be added to the dynamic database after validation and analysis.

## Technical Validation

Mutation identification methods are critical for data credibility, which is particularly important for drug resistance comparison, tracking, and lineage classification. To validate our data, we performed a similar analysis using a different approaches and two published pipelines MTBseq and TB-profiler to ensure that our generated dataset is accurate^13,26^. Due to the substantial number of samples in the dataset, technical validation was performed in a small batch. We randomly collected 271 SRAs belonging to all lineages in our datasets (**AFRO-TB Dataset Validation-strains**)^18^. MTBseq and TB-profiler, are pipelines that perform TB analyses including drug resistance identification and lineage classification using the same reference genome. MTBseq^26^ was used with default settings to map the fastq sequences to the reference genome *Mycobacterium tuberculosis H37Rv* (GenBank accession number NC_000962.3) using BWA-MEM. SAM files were then converted to BAM using SAMtools^27^. Mapping errors were corrected alongside recalibration of base calls using GATK and mpileup files were created using GATK^28^ to facilitate variant calling by SAMtools. Lineage classification is based on a set of phylogenetic SNPs. Similarly, TB-profiler was run with the fastq files of the validation samples with default settings.

**Fig. 3:**
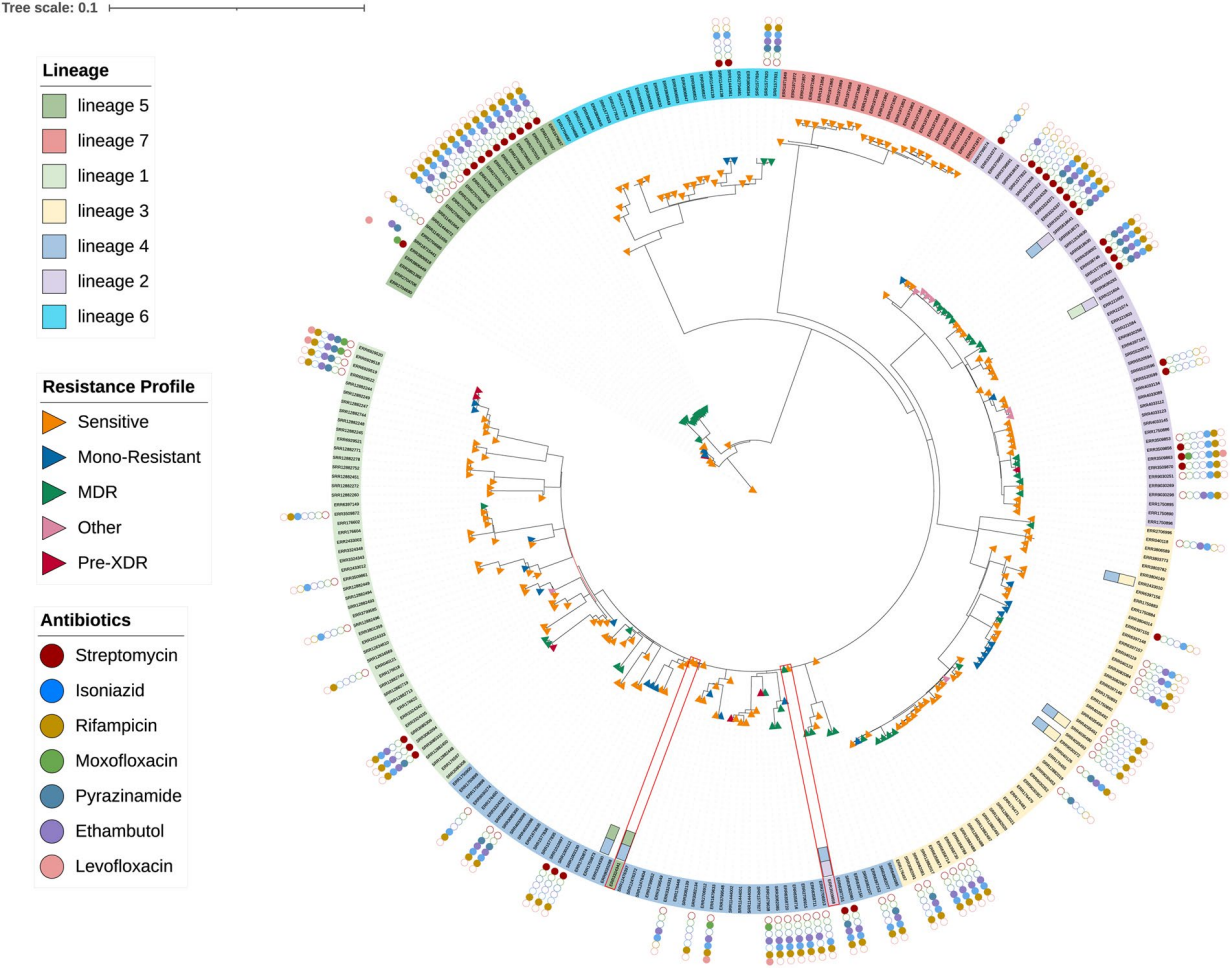
Phylogenetic tree constructed from 271 *Mycobacterium tuberculosis* strains from AFRO-TB and the validation dataset. The color of the labels represents the distribution of lineages. The rectangle represents the resistance classification. The small circles outside the phylogeny represent the antibiotics associated with the identified mutation. The empty circles represent the absence of mutations associated with the corresponding antibiotic, while the full circles represent the presence of the corresponding mutations. The red rectangle represents the discordance between the AFRO-TB and the validation dataset.The 8 rectangles inside the circle represent the TB-profiler inconclusive results, and the colors represent the suspected lineages.

Phylogenetic analysis was performed on the VCF files of the Afro-TB dataset using Nextstrain^29^, and comparative analysis was visualized using iTol^30^. Resistance analysis showed that the majority of strains (64%) were susceptible, 2.5% preXDR, 19.6% MDR, 10% monoresistance, and 3% have other resistance. The resistance results of the validation dataset are consistent between TB-profiler, MTBseq and Afro-TB (Fig. 3).

However, the lineage identification results in the Afro-TB dataset were more accurate than the validation dataset results using MTBseq and TB -profiler. MTBseq contains 2 misidentified strains, ERR3324341 and ERR3509858, which were assigned to lineages L5 and L2, respectively. These two strains were identified in the Afro-TB dataset as belonging to L4. These results were confirmed by phylogenetic tree analysis and the literature papers associated with these isolates^31^. TB-profiler, however, provided inconclusive results for 8 of 271 strains (Fig. 3). These results support the conclusion that the Afro-TB dataset is more accurate in assigning lineages than both MTBseq and TB-profiler. African countries have a high prevalence of TB. It is important that other regions of the world take action to prevent the spread of TB. Detailed analyses of genomic data and easy access to comparative and tracking tools will enable a better understanding of the genetics and transmission of drug-resistant TB, which could lead to more effective management of TB in clinical and public health settings.

## Usage Notes

African countries have a high prevalence of TB. It is important that other regions of the world also take action to prevent the spread of TB. Detailed analyses of genomic data and easy access to comparative and tracking tools will provide a better understanding of the genetics and transmission of drug-resistant TB, which could lead to more effective management of TB in clinical and public health settings.

**Fig. 4:**
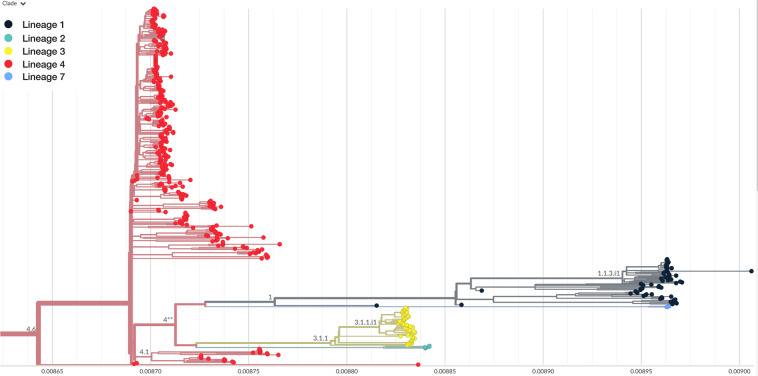
Phylogenetic tree of 500 *Mycobacterium tuberculosis* isolats using Nextstrain. The colors represent the different TB lineages.

The full dataset is available in a Figshare repository^18^ and at https://bioinformatics.um6p.ma/AfroTB/. This dataset can be used to study the evolution of TB in Africa. It facilitates analysis by providing researchers in different countries with a ready-to-use dataset to compare, assess, and track the source of outbreaks. This dataset could also be used for studies on resistance evolution in Africa and around the world. Subsequent phylogenetic analyses will benefit from a large number of labeled and preprocessed genomes. The mutation table can serve as a reference for comparative resistance analysis and the VCF files in AFRO-TB are ready to be used for Nextstrain analysis Fig. 4.

We used the generated VCF and curated metadata of 500 isolates to perform phylogenetic analysis as an example of application for our dataset. Based on different locations and lineage types, 500 VCFs were selected from the Afro- dataset. The selected VCFs were merged using bcftools and used as input to the augur software^32^ implemented in Nextstrain. Metadata for each sample was retrieved from the dataset and used for time tracking and geographic distribution visualization in Nextstrain (4).

## Data Availability

All programs used in this study were published in peer-reviewed journals. Additional information was detailed in the Materials and Methods section.
